# Plasma Methylmalonic Acid Concentration in Folic Acid–Supplemented Depressed Patients with Low or Marginal Vitamin B-12: A Randomized Trial

**DOI:** 10.1093/jn/nxab280

**Published:** 2021-09-11

**Authors:** Ben Carter, Zohra Zenasni, Stuart J Moat, Peter R Hudson, Ian T Russell, Andrew McCaddon, The FolATED group consists of the following authors, Rhiannon Whitaker, Joshua Pink, Seren Roberts, Clare Wilkinson, Dyfrig Hughes, Emma Betson, Diana Carr, Andrea Jorgenson, Munir Pirmohamed, Nevyn Williams, Helen Lewis, Keith Lloyd, Yvonne Sylvesture, Richard Tranter

**Affiliations:** Department of Biostatistics and Health Informatics, Institute of Psychiatry, Psychology and Neuroscience, King's College London, London, United Kingdom; Department of Biostatistics and Health Informatics, Institute of Psychiatry, Psychology and Neuroscience, King's College London, London, United Kingdom; School of Medicine, Cardiff University, University Hospital of Wales, Cardiff, United Kingdom; Department of Medical Biochemistry and Immunology & Toxicology, Cardiff and Vale University Health Board, Cardiff, United Kingdom; Cobalz Limited, Chester, United Kingdom; Swansea Trials Unit, Swansea University Medical School, Swansea, United Kingdom; Faculty of Social and Life Sciences, Wrexham Glyndwr University, Wrexham, United Kingdom; Bangor University; Bangor University; Bangor University; Bangor University; Bangor University; University of Liverpool; University of Liverpool; University of Liverpool; University of Liverpool; University of Liverpool; University of York; University of Swansea; University of Swansea; University of Otago

**Keywords:** folic acid, vitamin B-12, methylmalonic acid, metabolism, public health

## Abstract

**Background:**

Individuals with low serum vitamin B-12 and high serum folate have higher plasma concentrations of methylmalonic acid (MMA). Whether folic acid (FA) causes an increase in MMA is not known.

**Objectives:**

We aimed to determine the impact of FA supplementation on plasma MMA concentration in people with low or marginal serum vitamin B-12.

**Methods:**

We conducted a multicenter double-blind placebo-controlled randomized trial of oral FA (5 mg/d for 12 wk) in middle-aged patients treated with antidepressant medication participating in the FoLATED (Folate Augmentation of Treatment—Evaluation for Depression) trial. Participants defined as having “low” serum vitamin B-12 (vitamin B-12 ≥150 and <220 ng/L) or “marginal” serum vitamin B-12 (vitamin B-12 ≥ 220 and <280 ng/L) were included. The primary outcome of this substudy was MMA at week 12. A mixed-effects linear regression was fitted and reported using the adjusted mean difference (aMD).

**Results:**

A total of 177 participants were included (85 randomly assigned to placebo and 92 to FA); the mean ± SD age was 46.2 ± 11.8 y, and 112 (63.3%) were female. The MMA analysis included 135 participants and the aMD was −0.01 (95% CI: −0.06, 0.04; *P* = 0.71). Serum folate was measured on 166 participants and increased in the supplementation group; the aMD was 21.6 μg/L (95% CI: 8.13, 25.02 μg/L; *P *< 0.001). A total of 117 participants were assessed for RBC folate, which also increased in the supplementation group; the aMD was 461 μg/L (95% CI: 387, 535 μg/L; *P *< 0.001).

**Conclusions:**

Supplementation of FA leads to an increase of serum and RBC folate, but does not change plasma MMA concentration in individuals with serum vitamin B-12 between 150 and 280 ng/L. We cannot exclude effects in older people or those with serum vitamin B-12 <150 ng/L. Previously reported associations may arise from effects of impaired vitamin B-12 status on folate metabolism.

This trial was registered at www.isrctn.com as ISRCTN37558856.

## Introduction

The interaction between vitamin B-12 and folate has important public health implications. For example, folic acid (FA) supplementation corrects the anemia of vitamin B-12 deficiency but not vitamin B-12–related neurological changes (1). Permanent nerve damage might occur if vitamin B-12 deficiency remains untreated, although the evidence for such “masking” is scarce, even after folate fortification of food (2). The rationale for many countries to introduce such fortification was to reduce the incidence of neural tube defects (3), and this has proved remarkably successful (4). However, the combination of mandatory fortification and self-supplementation has exposed a significant proportion of these populations to concentrations of FA above the upper intake level (5).

This has led to various concerns regarding the potential toxicity of such exposure. These include possible associations with an increased incidence of colorectal and breast cancers, autism, and cognitive function (5), although significant controversy exists (6). For example, in areas with mandatory fortification, high serum folate combined with low serum vitamin B-12 is associated with cognitive impairment in the elderly (7–10). However, no such association is observed in nonfortified countries (11, 12).

A cross-sectional study of >10,000 participants in the US NHANES observed a relation between serum folate and a metabolic marker of an enzymatic function of vitamin B-12 (13). The vitamin B-12–dependent enzyme methylmalonyl CoA mutase converts methylmalonyl-CoA to succinyl-CoA. Plasma methylmalonic acid (MMA) concentration increases with suboptimal serum vitamin B-12 and, in the absence of renal impairment, is a useful functional marker of its status. However, in individuals with low serum vitamin B-12 (<148 pmol/L ∼ 200 ng/L) in the NHANES study, mean MMA concentration increased significantly with increasing serum folate (*P* = 0.008) (13).

There is little prior evidence that FA affects MMA accumulation and biologically one might not expect any interaction between these 2 molecules. However, several other cross-sectional reports have also suggested that this might occur in the context of high FA intake from mandatory fortification of foods plus high supplement usage (14). The question is relevant because it might provide evidence that high FA intake interferes with vitamin B-12 metabolism.

One hypothetical explanation is an adverse oxidative effect of unmetabolized FA (UMFA) on vitamin B-12 homeostasis (13). Alternatively, people with existing low vitamin B-12 might fail to synthesize polyglutamated intracellular folate—an essential prerequisite for its cellular retention—hence accounting for the observed cross-sectional association between MMA and serum folate (15).

There is a high prevalence of metabolic vitamin B-12 deficiency in the elderly (16). This is associated with lower cognitive function scores (17). If FA increases MMA concentration, this is vitally important for public health decisions concerning food fortification (6, 18).

It is impossible to distinguish cause from effect in cross-sectional studies (19, 20). We designed the FolATED (Folate Augmentation of Treatment—Evaluation for Depression) trial to investigate FA augmentation of antidepressive treatment by randomly assigning patients to either 5 mg of FA or placebo daily (21).

The objective of this substudy was to assess the effect of FA supplementation on MMA concentration in patients with low or marginal vitamin B-12 concentrations.

## Methods

### Patients

FolATED was a double-blind, placebo-controlled randomized trial conducted across 3 UK centers (22). Between July 2007 and November 2010 we recruited a sample of people with moderate to severe depression from primary or secondary care in 3 centers across Wales, United Kingdom. Of the 475 patients randomized to daily placebo or 5 mg FA daily for 12 wk, we included 440 in the FolATED analysis by treatment allocated. The substudy inclusion criteria were patients having low (150–219.9 ng/L) or marginal (220–280 ng/L) serum vitamin B-12 at recruitment. Blood samples were taken at baseline and at 12 wk.

### Settings

Three centers throughout Wales were involved with recruitment: North East Wales, North West Wales, and Swansea.

### Randomization

The sequence was generated using a varying permuted block design stratified by site.

### Laboratory methods

Serum and RBC folate and serum vitamin B-12 were analyzed at local National Health Service laboratories on the day of collection. “Access” folate and vitamin B-12 chemiluminescence immunoassays (Beckman Coulter) were used in North East and North West Wales, whereas “Elecsys” folate and vitamin B-12 electrochemiluminescence immunoassays (Roche) were used in Swansea. We undertook biochemical analyses in the Department of Medical Biochemistry at the University Hospital of Wales in Cardiff. The samples for homocysteine and MMA were stored frozen at −70°C until completion of the study. We measured plasma homocysteine using a commercially available assay for the Abbott Diagnostics ARCHITECT system, and plasma MMA using a GC-MS assay (23). To minimize interbatch variation, we assayed samples from the same participant in the same batch; the mean intrabatch CV was <3% for homocysteine and <8% for MMA.

### Power calculations

Fedosov (24) suggested that patients with vitamin B-12 as high as 280 ng/L may be vitamin B-12 deficient. We assayed MMA in all (*n* = 177) patients with baseline vitamin B-12 between 150 and 280 ng/L and estimated this would yield 80% power to detect an increase of 42% in mean MMA concentration after augmentation of FA, assuming there was an effect, using a type 1 error at 0.05.

### Ethics

In 2006, the Multicenter Research Ethics Committee (MREC) for Wales gave ethical approval for FolATED, and the Medicines and Healthcare products Regulatory Agency issued the Clinical Trial Authorization. In 2009 the MREC gave additional permission to investigate the interaction between FA and MMA.

### Outcomes

The primary outcome was MMA concentration at week 12. The secondary outcomes were serum folate, RBC folate, and serum vitamin B-12; and homocysteine collected at week 12.

### Outcome assessment timing and blinding status of participants

Blood samples were drawn from the included participants to assess the biochemistry outcomes at baseline and week 12. The participants, and researchers taking the sample, were blinded to the allocation. The analysts undertaking the initial analysis were pseudo-blinded during the FolATED analysis (22). All researchers were unblinded at the timing of the analysis for this study.

### Statistics

#### Data analysis

We followed the CONSORT reporting checklist for reporting a clinical trial.

Baseline characteristics (age, gender, antidepressant, counselling, marital status, number of children, employment status, smokers, alcohol consumption, low or marginal serum vitamin B-12, plasma MMA, serum folate, RBC folate, homocysteine) were summarized by randomized group. For continuous variables, we reported both the mean ± SD and median [IQR] values.

The primary and secondary outcomes were analyzed using a mixed-effects linear regression, adjusting for fixed effects of randomized group (FA or placebo) and baseline concentration, and with center as a random intercept to account for heterogeneity across sites. The adjusted mean differences (aMDs) are presented along with 95% CIs and *P* values. A complete-case population was used throughout and reasons for missingness were explored and used to determine any breaches of a missing-at-random assumption.

aMDs were converted to standardized effect sizes and presented alongside their associated 95% CI for each of the 5 outcomes. Stata version 15 (StataCorp.2017. College Station, TX: StataCorp LLC) was used throughout.

#### Sensitivity analysis

A sensitivity analysis was carried out to assess the impact of including patients with marginal vitamin B-12 at baseline. The analysis was repeated only including patients with low vitamin B-12 at baseline.

## Results

**FIGURE 1:**
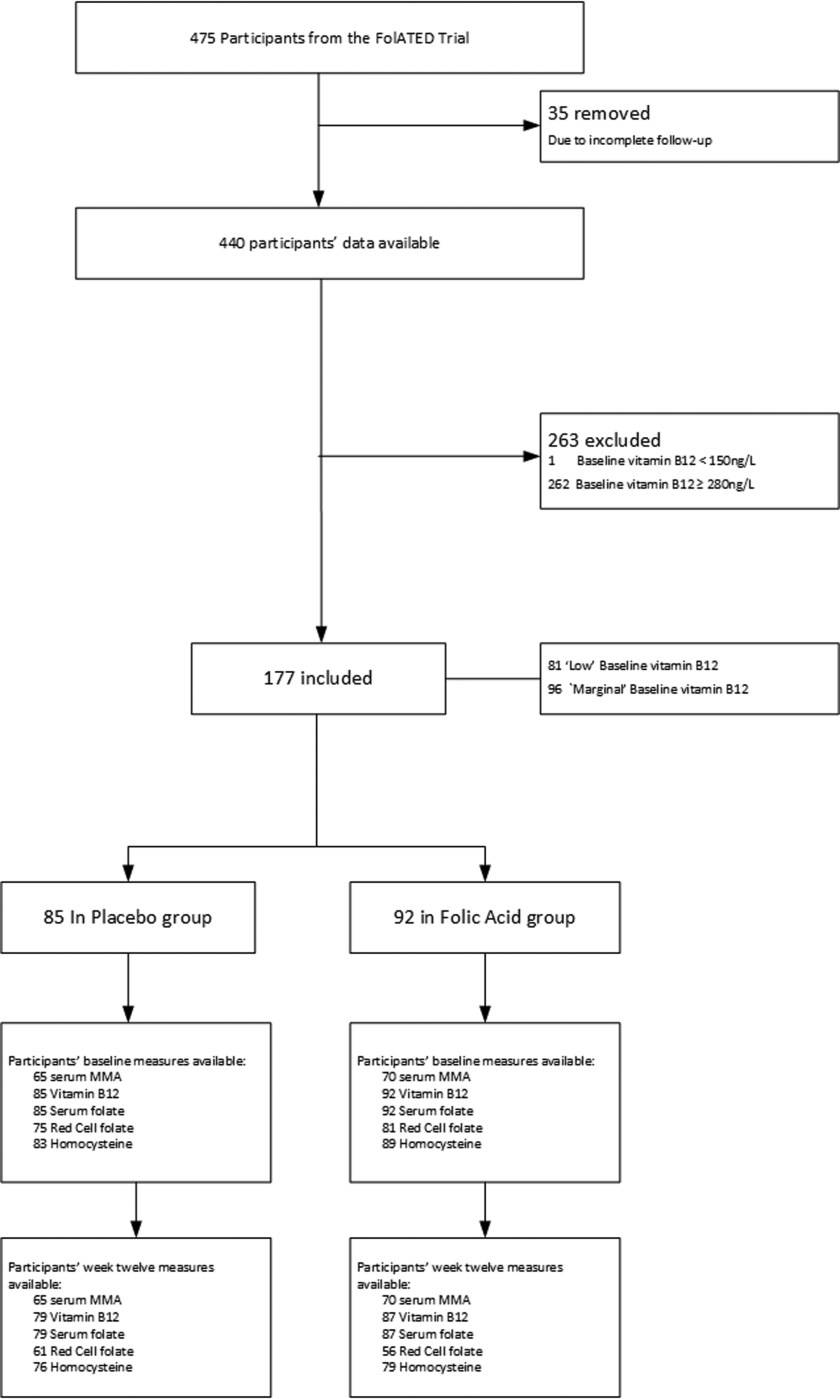
CONSORT diagram of participants included from the FolATED trial. FolATED, Folate Augmentation of Treatment—Evaluation for Depression; MMA, methylmalonic acid.

Of the 177 participants (**Figure 1**), **Table 1** shows 81 had “low” baseline serum vitamin B-12 between 150 and 220 ng/L (36 in the placebo group, 45 in the intervention group) and another 96 had “marginal” baseline concentrations between 220 and 280 ng/L (49 in the placebo group, 47 in the intervention group). The mean age was 46 y in the placebo group and 46.3 y in the FA group. There were 32 males (37.6%) in the placebo group and 33 males (35.9%) in the FA group; all baseline characteristics were similar across the 2 allocation groups.

**TABLE 1:** Baseline characteristics for participants with “low” (150–219.9 ng/L) or “marginal” (220–280 ng/L) serum vitamin B-12^1^

Baseline characteristics	Placebo (*n* = 85)	Folic acid (*n* = 92)	Total (*n* = 177)
Age, y			
	46.0 ± 11.6	46.3 ± 12.1	46.2 ± 11.8
	46.9 [38.6–54.0]	47.1 [38.5–54.0]	46.9 [38.6–54.0]
Gender			
Male	32 (37.6)	33 (35.9)	65 (36.7)
Female	53 (62.4)	59 (64.1)	112 (63.3)
Antidepressant			
SSRI	56 (65.9)	62 (67.4)	118 (66.7)
Other	29 (34.1)	30 (32.6)	59 (33.3)
Counselling			
Counselling	39 (45.9)	35 (38.0)	74 (41.8)
No counselling	46 (54.1)	57 (62.0)	103 (58.2)
Marital status			
Single	15 (17.6)	19 (20.7)	34 (19.2)
Had a partner	21 (24.7)	11 (12.0)	32 (18.1)
Have a partner	49 (57.6)	62 (67.4)	111 (62.7)
Dependent children, *n*			
0	52 (61.2)	58 (63.0)	110 (62.1)
1	13 (15.3)	11 (12.0)	24 (13.6)
2	12 (14.1)	13 (14.1)	25 (14.1)
≥3	8 (9.41)	10 (10.9)	18 (10.2)
Employment			
Full-time employed	29 (34.1)	17 (18.5)	46 (26.0)
Part-time employed	20 (23.5)	31 (33.7)	51 (28.8)
Unemployed	36 (42.4)	44 (47.8)	80 (45.2)
Smoking status			
Nonsmoker	54 (63.5)	59 (64.1)	113 (63.8)
Low (1–10/d)	16 (18.8)	11 (12.0)	27 (15.3)
Moderate (11–20/d)	11 (12.9)	17 (18.5)	28 (15.8)
High (≥21/d)	4 (4.71)	5 (5.44)	9 (5.09)
Alcohol consumption per week^2^			
None	33 (38.8)	37 (40.2)	70 (39.5)
Within safe limit	41 (48.2)	43 (46.7)	84 (47.5)
Above safe limit	11 (12.9)	12 (13.0)	23 (13.0)
Serum vitamin B-12, ng/L			
Low (150–219.9)	36 (42.4)	45 (48.9)	81 (45.8)
Marginal (220–279)	49 (57.6)	47 (51.1)	96 (54.2)

1 Values are *n*, mean ± SD, or median [IQR]. SSRI, selective serotonin reuptake inhibitor.

2 Safe limits: females = 14 units/wk; males = 21 units/wk.

Among participants with a low or marginal baseline serum vitamin B-12 in the FolATED trial, 5 mg FA/d increased mean serum and RBC folate in those allocated to receive it, from 5.8 μg/L and 383 μg/L at baseline to 27.9 μg/L and 830 μg/L at week 12, respectively (**Table 2**).

**TABLE 2:** Baseline and week 12 outcome measurements by treatment group^1^

	Baseline		Week 12	
	Placebo (*n* = 85)	FA (*n* = 92)	Placebo (*n* = 79)	FA (*n* = 87)
Vitamin B-12, ng/L				
	226 ± 35.0	218 ± 36.2	234 ± 66.6	236 ± 61.2
	230 [199–258]	221 [189–248]	222 [189–262]	227 [196–266]
Serum folate, μg/L				
	6.54 ± 3.81	5.80 ± 3.11	7.53 ± 6.91	27.9 ± 15.3
	5.70 [3.70–9.00]	5.10 [3.55–6.85]	5.80 [3.50–9.00]	25.1 [18.0–36.9]
Plasma MMA, μmol/L	65	70	65	70
	0.313 ± 0.165	0.302 ± 0.160	0.313 ± 0.159	0.300 ± 0.156
	0.270 [0.190–0.340]	0.250 [0.180–0.360]	0.260 [0.200–0.390]	0.255 [0.190–0.350]
RBC folate, μg/L	75	81	61	56
	380 ± 160	383 ± 171	366 ± 153	830 ± 262
	343 [261–478]	337 [253–445]	327 [255–471]	925 [612–1040]
Homocysteine, μmol/L	83	89	76	79
	12.8 ± 4.11	14.6 ± 10.4	12.6 ± 3.89	11.2 ± 9.25
	11.9 [10.2–14.7]	12.8 [10.9–14.8]	11.9 [9.80–14.8]	10.0 [8.90–11.4]

1 Values are *n*, mean ± SD, or median [IQR]. FA, folic acid; MMA, methylmalonic acid.

### Primary outcome analysis

There was no difference in the between-group plasma MMA concentration; the aMD was −0.01 (95% CI: −0.06, 0.04; *P* = 0.71).

### Secondary outcome analyses

**FIGURE 2:**
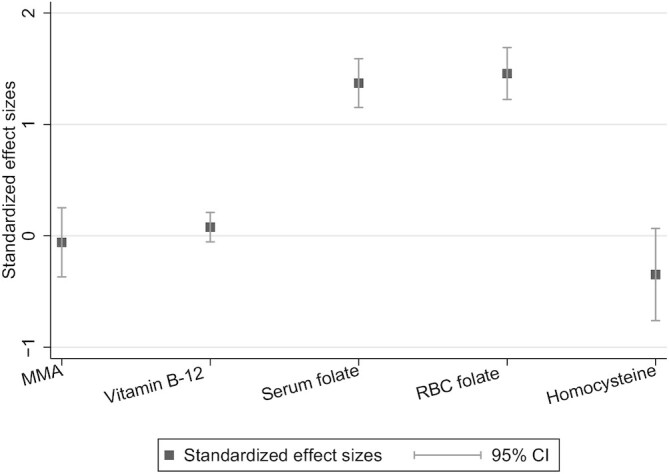
Results from the mixed-effects linear regression presenting the adjusted mean differences (with associated 95% CIs) for the biochemical outcomes. MMA, methylmalonic acid.

There was a strong statistically significant difference reported at week 12 in serum folate (aMD: 21.6 μg/L; 95% CI: 18.1, 25.0 μg/L; *P *< 0.001) and RBC folate (aMD: 461 μg/L; 95% CI: 388, 535 μg/L; *P *< 0.001), with greater concentrations in the supplemented group than in the control group. There was no difference found in homocysteine (aMD: −1.83 μmol/L; 95% CI: −4.01, 0.35 μmol/L; *P* = 0.10) or serum vitamin B-12 (aMD: 9.63 μg/L; 95% CI: −6.71, 26.0 μg/L; *P*  = 0.25) **Table 3**. However, caution is needed when interpreting the effects on serum homocysteine and serum vitamin B-12 because these analyses may be underpowered. All biochemical findings are shown as standardized effect sizes to facilitate comparison (**Figure 2**).

The missing data within the biochemistry outcomes were explored and found to be due to the facilities’ inability to collect and record all data. For example, 1 center was not able to collect or process MMA or RBC folate data, thus we judged these as missing completely at random (**Table 4**).

To determine whether an effect was concentrated within a more inadequate vitamin B-12 status range, a subset analysis of the 81 participants with vitamin B-12 <220 ng/L was performed as a secondary sensitivity analysis. Within the sample with a “low” vitamin B-12 baseline measure the between-group MMA aMD was 0.01 (95% CI: −0.07, 0.09; *P* = 0.75), i.e., comparing the supplemented group with the control group. Similar findings were reported for each of the biochemistry measures, thus a low vitamin B-12 patient population exhibited similar findings to those from a low or marginal population.

## Discussion

The FolATED trial showed conclusively that FA does not potentiate antidepressant medication (22). In this subgroup of depressed but otherwise healthy participants with baseline serum vitamin B-12 concentrations that were “low” (between 150 and 220 ng/L) or “marginal” (between 220 and 280 ng/L), 5 mg FA/d for 12 wk did not significantly change MMA concentration.

Our study has several strengths. We believe this is the first reported randomized intervention study of FA in patients with low or marginal serum vitamin B-12, and the first to examine the hypothesis that FA affects MMA concentration in such individuals; this has not previously been challenged or adequately replicated in the literature.

The fact that patients received FA alone in the FoLATED trial makes it unique; in most other trials investigating FA, participants are treated with a combination of FA plus vitamin B-12 in order to avoid alleged adverse effects from FA.

It is also important to note that our study participants had an average age highly similar to that of the NHANES study (13). The concentrations of serum folate achieved in our study were at least as high as those seen in previous studies and homocysteine was lower after 12 wk in the group receiving FA therapy; daily supplementation with 5 mg FA is expected to reduce homocysteine blood concentration by approximately one-quarter, according to our study's baseline values (i.e., from 14.6 μmol/L to 11 μmol/L) (25). Consistent with this, homocysteine concentrations fell from baseline in the FA-supplemented group to a mean of 11.2 μmol/L, although there was no statistical difference in the week 12 between-group mean difference (*P* = 0.10). Nevertheless, this fall, together with the very high serum folate and RBC folate concentrations achieved, does mean that biologically relevant metabolites are altered by FA treatment, making the fact that there is absolutely no change in MMA very interesting.

However, there are several important weaknesses. First, we powered the FolATED trial to test whether FA potentiates antidepressant medication. Because that limits power to investigate MMA in low serum vitamin B-12 we set the criterion for that at 220 ng/L rather than 200 ng/L (13); we also extended MMA assays to those with “marginal” vitamin B-12, because Fedosov (24) suggested they might also have a vitamin B-12 insufficiency. Secondly, the exclusion criteria for FolATED included a serum vitamin B-12 concentration <150 ng/L. Hence, we cannot exclude the possibility that FA increases MMA at *very* low serum vitamin B-12 concentrations. However, ethical constraints on administering FA to vitamin B-12–deficient patients will prevent any such trial being performed (6, 20). We also cannot completely exclude the possibility of a potential drug (antidepressant) and nutrient (FA) interaction, although this would seem unlikely. Finally, the FolATED trial population had a mean age of 46 y. Hence, we cannot exclude the possibility that FA increases MMA in older people. It is also possible that a longer exposure to FA might adversely influence MMA concentrations.

Nevertheless, we found no evidence of an effect of high daily doses of FA over 3 months on MMA concentrations. Others have suggested that the association observed in cross-sectional studies might be due to a direct adverse effect of UMFA on vitamin B-12 metabolism (13). Unfortunately, we did not directly measure UMFA in our study. Our findings perhaps lend support to the alternative interpretation: that an elevated folate concentration is a consequence of pre-existing vitamin B-12 deficiency. This could arise either from impaired cellular uptake of folate or from its increased efflux in vitamin B-12 deficiency. There are no known mechanisms for the former. The latter suggestion relates to the “methyl-folate trap” whereby reduced conversion of 5-methyl-tetrahydrofolate (5mTHF) to tetrahydrofolate, owing to reduced activity of the vitamin B-12–dependent enzyme methionine synthase, leads to egress of unconjugated 5mTHF into the circulation because 5mTHF is not a preferred substrate for folyl polyglutamate synthase (15, 26). Although this might indeed explain the observed cross-sectional association between MMA and serum folate it is inconsistent with the increase in RBC folate seen in our FA-supplemented patients (Tables 2, 3) and with the observation by Miller et al. (19) of high RBC folate associated with raised MMA in elderly vitamin B-12–deficient individuals.

**TABLE 3:** Mixed-effects regression of the biochemical outcomes, presenting the crude MD and aMD for folic acid supplementation – placebo^1^

	MD (95% CI)	*P* value	aMD (95% CI)	*P* value
Plasma MMA concentration, μmol/L	−0.0131 (−0.0663, 0.0401)	0.63	−0.00909 (−0.0673, 0.0392)	0.71
Vitamin B-12, ng/L	1.91 (−17.5, 21.4)	0.85	9.63 (−6.71, 26.0)	0.25
Serum folate, μg/L	20.2 (16.6, 23.8)	<0.01	21.6 (18.1, 25.0)	<0.01
RBC folate, μg/L	464 (387, 541)	<0.01	461 (387, 535)	<0.01
Homocysteine, μmol/L	−1.39 (−3.64, 0.856)	0.23	−1.83 (−4.01, 0.352)	0.10

1 aMD, adjusted mean difference; MD, mean difference; MMA, methylmalonic acid.

**TABLE 4:** Missing observations for each outcome by randomized group^1^

Measure	Placebo (*n* = 85)	Folic acid (*n* = 92)
At baseline		
Vitamin B-12	0 (0.0)	0 (0.0)
Plasma MMA concentration	20 (23.5)	22 (23.9)
Serum folate	0 (0.0)	0 (0.0)
RBC folate	10 (11.8)	11 (12.0)
Homocysteine	2 (2.35)	3 (3.26)
At 12 wk		
Vitamin B-12	6 (7.06)	5 (5.44)
Plasma MMA concentration	20 (23.5)	22 (23.9)
Serum folate	6 (7.06)	5 (5.44)
RBC folate	24 (28.2)	36 (39.1)
Homocysteine	9 (10.6)	13 (14.1)

1 Values are *n* (%). MMA, methylmalonic acid.

In 1 retrospective review the association between MMA and folate occurred only in participants >60 y old with “low to normal” serum vitamin B-12 (27). No association was apparent in a group of university students with low serum vitamin B-12 (28). The mean age of our participants was 46 y, hence we cannot exclude the possibility that FA affects MMA in older populations. Perhaps the association becomes more apparent in the elderly, as age-related renal impairment slows MMA excretion (29, 30).

These findings address an important public health issue (20) of the impact of FA augmentation. FA increases RBC folate and serum folate, but supplementation does not change plasma MMA concentration in middle-aged individuals with serum vitamin B-12 between 150 and 280 ng/L. With these constraints in mind, we suggest that FA does not affect biological pathways leading to adverse health effects, nor vitamin B-12’s function and its role in sustaining health (6), at least in young and middle-aged subjects.

In conclusion, augmentation of FA did lead to a change in RBC folate and serum folate but did not lead to any change in MMA in patients with low or marginal serum vitamin B-12. Further interventions, with a greater sample size and longer duration of treatment, are ultimately required to determine whether any biological interaction exists between FA intake and serum MMA concentration.

## Data Availability

Data described in the article, code book, and analytic code will be made available upon request pending application and approval of an a priori statistical analysis plan that addresses a scientifically justified research question and is approved by the chief investigator.

## Notes

The FolATED (Folate Augmentation of Treatment—Evaluation for Depression) trial (21) was supported by UK National Institute of Health Research Health Technology Assessment programme project number 04/35/08 (to ITR). The funder played no role in designing and implementing the trial or analyzing and interpreting data; the full trial was published in *Health Technology Assessment* 2014;18(48) (22).

Author disclosures: AM and PRH are scientific advisors to, and shareholders of, Cobalz Limited, a private limited company developing novel B-vitamin and antioxidant supplements. All other authors report no conflicts of interest.

Abbreviations used: aMD, adjusted mean difference; FA, folic acid; FolATED, Folate Augmentation of Treatment—Evaluation for Depression; MMA, methylmalonic acid; MREC, Multicenter Research Ethics Committee; UMFA, unmetabolized folic acid; 5mTHF, 5-methyl-tetrahydrofolate.
